# ﻿On the verge of extinction – revision of a highly endangered Swiss alpine snail with description of a new genus, *Raeticella* gen. nov. (Gastropoda, Eupulmonata, Hygromiidae)

**DOI:** 10.3897/zookeys.1104.82866

**Published:** 2022-06-08

**Authors:** Jeannette Kneubühler, Markus Baggenstos, Eike Neubert

**Affiliations:** 1 Natural History Museum Bern, 3005 Bern, Switzerland Natural History Museum Bern Bern Switzerland; 2 Institute of Ecology and Evolution, University of Bern, 3012 Bern, Switzerland University of Bern Bern Switzerland; 3 Oekologische Beratung Markus Baggenstos, Tottikonstrasse 48, 6370 Stans, Switzerland Oekologische Beratung Markus Baggenstos Stans Switzerland

**Keywords:** Endemism, integrative taxonomy, LGM, mountains, nunataks, phylogeny, Switzerland, *Trochulus*

## Abstract

The phylogenetic status of the alpine land snail *Fruticicolabiconica* has remained questionable since it was described by Eder in 1917. Considered a microendemic species from mountain tops in Central Switzerland, the shell is specially adapted for life under stones. Herein, we show via molecular and anatomical investigations that *F.biconica* neither belongs to the land snail genus *Trochulus*, nor to any other genus within Trochulini, but rather warrants placement within the newly established genus *Raeticella* Kneubühler, Baggenstos & Neubert, 2022. Phylogenetic analyses reveal that *R.biconica* is clearly separated from *Trochulus*. These findings are supported by morphological investigations of the shell and genitalia.

## ﻿Introduction

Discovered in the Bannalp, Nidwalden and known from only a few localities in the Central Swiss Alps, *Fruticicolabiconica* was described by the Swiss zoologist Leo Eder in 1917.

Later, *F.biconica*, known as the Nidwaldner hairy snail, was moved to the widely used genus *Trichia* W. Hartmann, 1840 and circulated throughout the European literature under this designation (e.g., Kerney et al. 1983). The generic name, *Trichia*, was subsequently replaced by *Trochulus* Chemnitz, 1786 due to homonymy with *Trichia* De Haan, 1839 (Crustacea, Xanthidae).

Previous studies (Pfenninger et al. 2005; Dépraz et al. 2009; Duda et al. 2014; Kruckenhauser et al. 2014; Proćków et al. 2021) included *T.biconicus* individuals in their genetic analyses of *Trochulus* species. Pfenninger et al. (2005) and Dépraz et al. (2009) used the same sequence of *T.biconicus* collected at the type locality at Bannalp. This sequence clustered within the so far known *Trochulus* species and some newly identified lineages, which were not further described (fig. 2 in Pfenninger et al. 2005; fig. 1 in Dépraz et al. 2009). Most likely, Pfenninger et al. (2005) and Dépraz et al. (2009) used misidentified specimens in their phylogenetic studies, or some samples were mixed. Since these authors did not publish images of the investigated specimens, an unequivocal identification is not possible. Duda et al. (2014) and Kruckenhauser et al. (2014) found that *T.biconicus*, “*T.oreinosoreinos*” (A.J. Wagner, 1915), and “*T.oreinosscheerpeltzi*” (Mikula, 1957) form basal lineages in comparison to specimens of *Trochulus* s. str. The latter two taxa were elevated from subspecies to species level (Bamberger et al. 2020) and are today known to belong to the newly described genus *Noricella* (Neiber et al. 2017). Proćków et al. (2021) found the same phylogenetic pattern as Duda et al. (2014) and Kruckenhauser et al. (2014) and questioned the affiliation of *biconicus* to *Trochulus*. Already Turner et al. (1998) had disputed the phylogenetic position of *T.biconicus*. Until today, the phylogenetic position of *T.biconicus* within the Trochulini remained unclear. Hence, an integrative taxonomic approach is applied in this study to investigate the phylogenetic affiliation of *T.biconicus*.

## ﻿Materials and methods

### ﻿Specimens investigated

**Figure 1.**
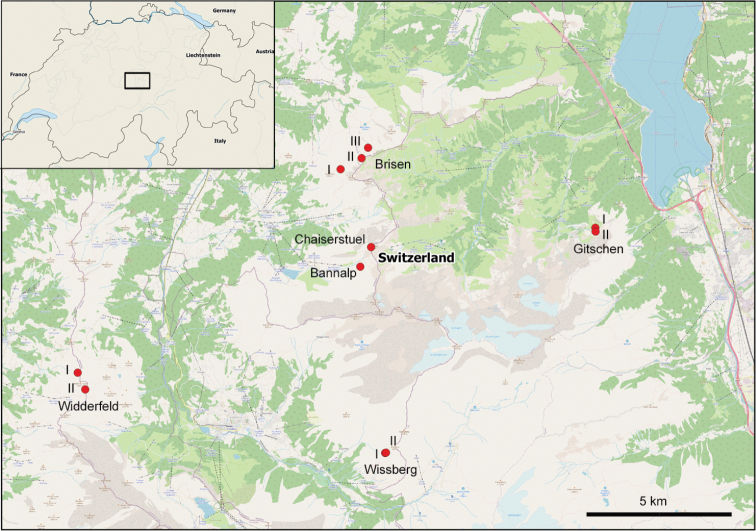
Sampling locations of the investigated individuals of *T.biconicus*.

Living individuals of *T.biconicus* were collected in September 2020 at 11 sites of the known distribution area in Central Switzerland (see Fig. 1 for detailed sampling localities). *Trochulusbiconicus* is classified as Vulnerable by Swiss law (Federal Office of Environment) and is protected. It is also considered Endangered by the IUCN (https://www.iucnredlist.org/species/22107/9360310). Collecting permits were obtained from the cantonal administrations of Nidwalden, Obwalden, and Uri. At each site, 3–5 snails were collected from large populations (>20 individuals) from under rocks on stony outcrops. The individual snails were preserved in 80% ethanol to keep the body tissue soft for proper anatomical investigations and DNA extraction. In Table 1, sampling localities and GenBank accession numbers are listed for all sequenced specimens of *T.biconicus*, *Trochulus* spp., and *Edentiellaedentula*. Usually, two specimens of *T.biconicus* per population were sequenced. Those not destroyed in the extraction process are deposited at the NMBE as voucher material. The map was produced with QGIS (2016, v. 2.18.13) using the Natural Earth data set.

**Table 1.** Sequenced *T.biconicus* specimens from Central Switzerland. Asterisk (*) marks the type localities of the species studied. Additionally, *Edentiellaedentula* (Draparnaud, 1805) and some species of *Trochulus* were sequenced and included for phylogenetic analyses.

Voucher-No.	Species	Locality	Coordinates	Altitude [m]	GenBank accession number COI	GenBank accession number 16S	GenBank accession number ITS2
NMBE 567164	*T.biconicus*	Bannalp Schonegg*	46.87°N, 8.46°E	2232	MW435154	MW433778	MW433799
NMBE 567165	*T.biconicus*	Bannalp Schonegg*	46.87°N, 8.46°E	2232	MW435155	MW433779	MW433800
NMBE 567167	*T.biconicus*	Chaiserstuel	46.87°N, 8.46°E	2263	MW435156	MW433780	MW433801
NMBE 567168	*T.biconicus*	Chaiserstuel	46.87°N, 8.46°E	2263	MW435157	MW433781	MW433802
NMBE 567149	*T.biconicus*	Wissberg I	46.81°N, 8.47°E	2335	MW435158	MW433782	MW433803
NMBE 567150	*T.biconicus*	Wissberg I	46.81°N, 8.47°E	2335	MW435159	MW433783	MW433804
NMBE 567152	*T.biconicus*	Wissberg II	46.81°N, 8.47°E	2355	MW435160	MW433784	MW433805
NMBE 567153	*T.biconicus*	Wissberg II	46.81°N, 8.47°E	2355	MW435161	MW433785	MW433806
NMBE 567155	*T.biconicus*	Widderfeld I	46.83°N, 8.33°E	2120	MW435162	MW433786	MW433807
NMBE 567156	*T.biconicus*	Widderfeld I	46.83°N, 8.33°E	2120	MW435163	MW433787	MW433808
NMBE 567159	*T.biconicus*	Widderfeld II	46.83°N, 8.33°E	2290	MW435164	MW433788	MW433809
NMBE 567161	*T.biconicus*	Brisen I	46.90°N, 8.45°E	2045	MW435165	MW433789	MW433810
NMBE 567137	*T.biconicus*	Brisen I	46.90°N, 8.45°E	2045	MW435166	MW433790	MW433811
NMBE 567139	*T.biconicus*	Brisen II	46.90°N, 8.46°E	2130	MW435167	MW433791	MW433812
NMBE 567140	*T.biconicus*	Brisen II	46.90°N, 8.46°E	2130	MW435168	MW433792	MW433813
NMBE 567142	*T.biconicus*	Brisen III	46.90°N, 8.46°E	2090	MW435169	MW433793	MW433814
NMBE 567143	*T.biconicus*	Brisen III	46.90°N, 8.46°E	2090	MW435170	MW433794	MW433815
NMBE 567145	*T.biconicus*	Gitschen I	46.88°N, 8.57°E	1890	MW435171	MW433795	MW433816
NMBE 567146	*T.biconicus*	Gitschen I	46.88°N, 8.57°E	1890	MW435172	MW433796	MW433817
NMBE 567148	*T.biconicus*	Gitschen II	46.88°N, 8.57°E	1970	MW435173	MW433797	MW433818
NMBE 567162	*T.biconicus*	Gitschen II	46.88°N, 8.57°E	1970	MW435174	MW433798	MW433819
NMBE 568100	*T.hispidus*	Sweden, prov. Uppland, Uppsala, Linnaeus Garden*	59.8619°N, 17.6342°E		ON477947	–	–
NMBE 568103	*T.hispidus*	Sweden, Östergötland, Vist	58.3294°N, 15.729°E	70	ON477948	ON479908	ON479901
NMBE 564609	*Trochulus* sp.	Bullet, Le Chasseron	46.8517°N, 6.5377°E	1606	ON477944	ON479905	ON479898
NMBE 564607	*Trochulus* sp.	Mervelier, Scheltental	47.336°N, 7.5153°E	615	ON477943	ON479904	ON479897
NMBE 543063	*Trochulus* sp.	St-Cergue, Route de Cuvaloup	46.4487°N, 6.123°E	1208	ON477941	ON479902	ON479895
NMBE 564601	*Trochulus* sp.	Zernez	46.6998°N, 10.0943°E	1473	ON477942	ON479903	ON479896
NMBE 568094	*Trochulus* sp.	Lac du Mont d’Orge	46.2321°N, 7.333°E	624	ON477946	ON479907	ON479900
NMBE 565821	*T.alpicola*	Bannalp Schonegg*	46.8706°N, 8.2491°E	2234	ON477945	ON479906	ON479899
MNHW_S_15_29_101	*T.villosus*	Montagne De Cernier	47.0763°N, 6.8888°E	1385	MW440985	MW447773	MW440678
MNHW_S_15_29_02	*T.clandestinus*	Montagne De Cernier	47.0763°N, 6.8888°E	1385	MW440983	MW447772	MW440676
MNHW_S_15_27_12	*Trochulus* sp.	Gorges de Court	47.2553°N, 7.3439°E	608	MW440984	MW621002	MW440677
MNHW_S_15_21_02	*T.caelatus*	Gorges de Moutier*	47.2856°N, 7.3819°E	477	MW440982	MW621001	MW440675
MNHW_S_Er_50	*E.edentula*	Erschwil	47.3673°N, 7.555°E	459	MW440986	MW621003	MW440679
MNHW_S_Er_51	*E.edentula*	Erschwil	47.3673°N, 7.555°E	459	MW440987	MW621004	MW440680

### ﻿Acronyms of collections

**NMBE**Natural History Museum Bern, Switzerland;

**MNHW** Museum of Natural History Wrocław, University of Wrocław, Poland.

### ﻿Shell morphology and anatomical study of the genitalia

One animal was selected from each population for investigations of the shell morphology and the genital organs. The dissection of the genitalia was performed under a Leica MZ12 stereomicroscope using thin tweezers. The genital organs were removed from the body, spread on a wax-lined bowl and properly pinned with small needles. The total length of the situs was measured using Mitutoyo callipers. Proportions between different parts of the genitalia were estimated using the total situs length as a reference. Additionally, the inner structures of the penis and the penial papilla were investigated. Pictures of the situs and the shells were taken with a Leica M205 microscope camera using an image-processing program (Leica LAS X v. 3.6.0.20104, Switzerland). The shells were imaged in frontal, lateral, apical, and ventral position. Shell height and shell width were measured using the callipers to assess perpendicularity with the shell axis.

### ﻿Abbreviations used in the anatomical descriptions and figures

**AG** albumen gland;

**BC** bursa copulatrix;

**DS** dart sacs;

**Ep** epiphallus;

**Fl**flagellum;

**HD** hermaphroditic duct;

**MG** mucous glands;

**Pe** penis;

**PP** penial papilla;

**sh** shell height;

**sw** shell width;

**Va** vagina;

**VD** vas deferens.

### ﻿DNA extraction, PCR amplification and sequence determination

For total DNA extraction of the specimens, the Qiagen Blood and Tissue Kit (Qiagen; Hilden, Germany) was used in combination with a QIAcube extraction robot. Circa 0.5 cm^3^ of tissue was cut and placed in a mixture of 180 µl ATL buffer and 20 µl Proteinase K. It was then incubated for ca. 4 hours at 56 °C in a heater (Labnet, Vortemp 56, witec AG, Littau, Switzerland). For subsequent DNA extraction, the QIAcube extraction robot was used with the Protocol 430 (DNeasy Blood Tissue and Rodent tails Standard). In this study, two mitochondrial markers (COI and 16S) and one nuclear marker (5.8S rRNA+ITS2) were investigated. PCR mixtures consisted of 12.5 µl GoTaq G2 HotStart Green Master Mix (Promega M7423), 4.5 µl ddH_2_O, 2 µl forward and reverse primer each, and 4 µl DNA template. The primer pairs implemented for the PCR are listed in Table 2. The following PCR cycles were used: for COI, 2 min at 94 °C, followed by 40 cycles of 1 min at 95 °C, 1 min at 47 °C and 1 min at 72 °C and finally, 5 min at 72 °C; for 16S, 3 min at 96 °C, followed by 40 cycles of 30 s at 94 °C, 30 s at 50 °C and 30 s at 72 °C, and finally, 1 min at 72 °C; and for 5.8S rRNA+ITS2, 3 min at 94 °C, followed by 40 cycles of 30 s at 94 °C, 30 s at 50 °C and 30 s at 72 °C, and finally, 5 min at 72 °C (SensoQuest Tabcyclet and Techne TC-512, witec AG, Littau, Switzerland). The purification and sequencing of the PCR product was performed by LGC (LGC Genomics Berlin, Germany).

**Table 2.** Primer pairs used for PCR.

Gene	Primer	Sequence	Sequence length (bp)	Reference
COI	LCO1490	5′-GGTCAACAAATCATAAAGATATTGG-3′	655	Folmer et al. 1994
	HCO2198	5′-TAAACTTCAGGGTGACCAAAAAATCA-3′		
16S	16S cs1	5′-AAACATACCTTTTGCATAATGG-3′	440	Chiba 1999
	16S cs2	5′-AGAAACTGACCTGGCTTACG-3′		
ITS2	ITS2 LSU1	5′-GCTTGCGGAGAATTAATGTGAA-3′	900	Wade and Mordan 2000
	ITS2 LSU3	5′-GGTACCTTGTTCGCTATCGGA-3′		

### ﻿Phylogenetic analyses

The phylogenetic analyses were conducted using sequences obtained from GenBank and from this study, which were included as outgroup: *Ichnusotrichaberninii* Giusti & Manganelli, 1987, *Plicuterialubomirskii* (Ślósarski, 1881), *Petasinaunidentata* (Draparnaud, 1805), *Noricellaoreinos* (A.J. Wagner, 1915), *Noricellascheerpeltzi* (Mikula, 1957) (GenBank numbers and sampling localities published by Neiber et al. 2017), *Edentiellaedentula* (Draparnaud, 1805), and several ingroup specimens of *Trochulus* (Table 1). These species were selected to identify the phylogenetic position of *T.biconicus*.

For sequence processing and editing, the software package Geneious v. 9.1.8 (Biomatters Ltd) was used. Topologies were estimated using two different phylogenetic methods: Bayesian Inference (BI) and Maximum Likelihood (ML). Bayesian Inference was performed using Mr. Bayes v. 3.2.6 x64 (Huelsenbeck and Ronquist 2001; Ronquist and Huelsenbeck 2003; Altekar et al. 2004) via the HPC cluster from the University of Bern (http://www.id.unibe.ch/hpc). Evolutionary models for each subset were set to mixed models. The Monte Carlo Markov Chain (MCMC) parameter was set as follows: starting with four chains and four separate runs for 20 million generations with a tree sampling frequency of 1000 and a burn in of 25%. RAxML plug-in for Geneious (Stamatakis 2014) was implemented for computing ML inference, using Geneious’ plug-in with rapid bootstrapping setting, the search for the best scoring ML tree and 1000 bootstrapping replicates. The model, GTR CAT I was implemented.

## ﻿Results

### ﻿Phylogenetic analyses

**Figure 2.**
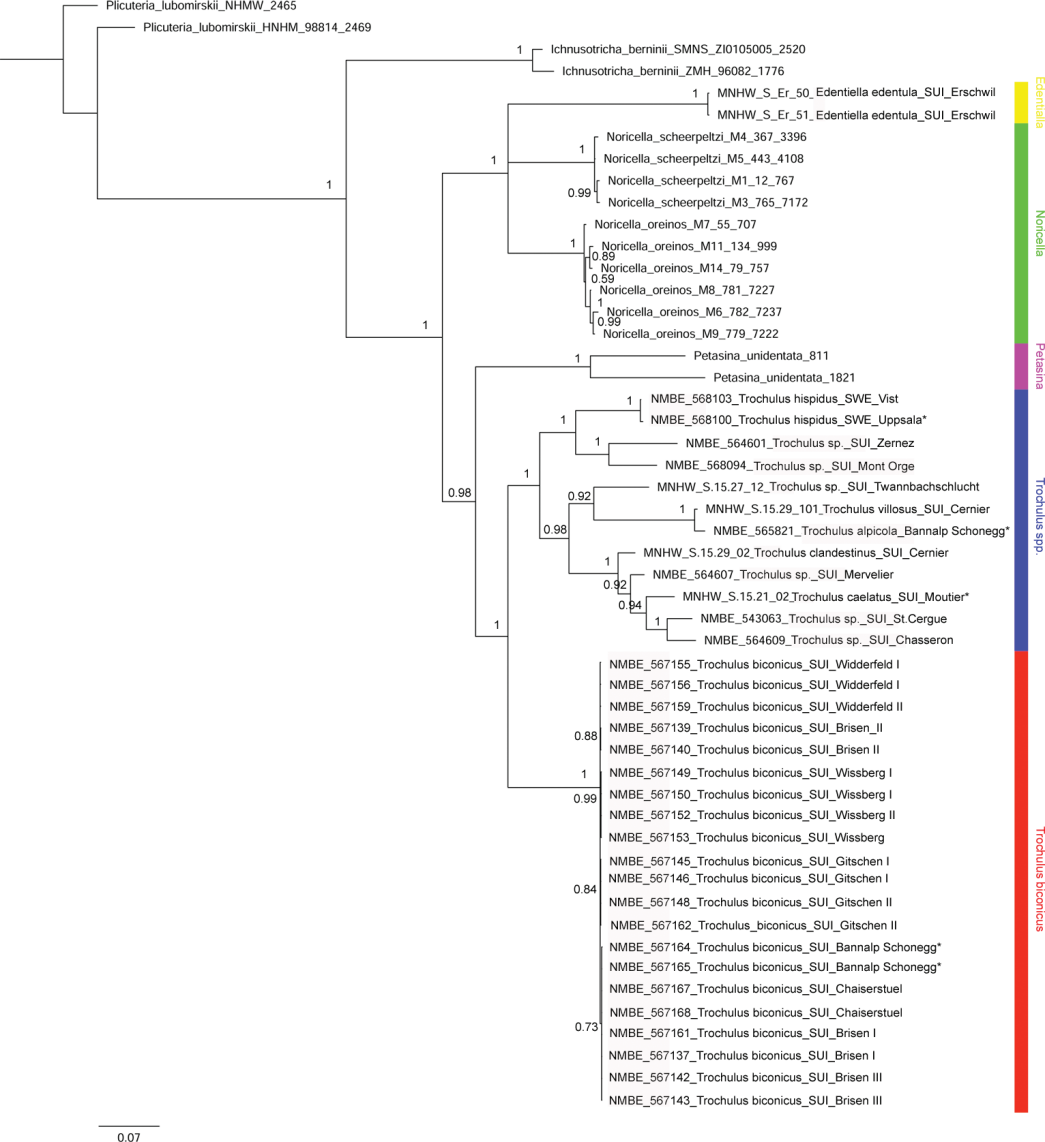
Bayesian Inference (BI) tree based on the concatenated data set of COI, 16S, and 5.8S rRNA+ITS2. Numbers represent Bayesian posterior probabilities.

The BI analysis of the concatenated data set (Fig. 2) shows two major clades within the tribe Trochulini. These two clades are separated with full support. One clade contains representative specimens of *Edentiella* and *Noricella* which form a polytomy. The second major clade within Trochulini contains representatives of *Petasina*, *Trochulus*, and the investigated *T.biconicus* specimens. *Trochulusbiconicus* is the sister lineage to the selected *Trochulus* specimens. This node has full posterior probability support. *Trochulushispidus* from the type locality in Sweden clusters together with a second specimen from Sweden and forms the sister group to two Swiss *Trochulus* specimens from Zernez and Lac du Mont d’Orge. The resolution within the *T.biconicus* clade is moderate because the investigated individuals differ in only few nucleotides in all three investigated markers.

**Figure 3.**
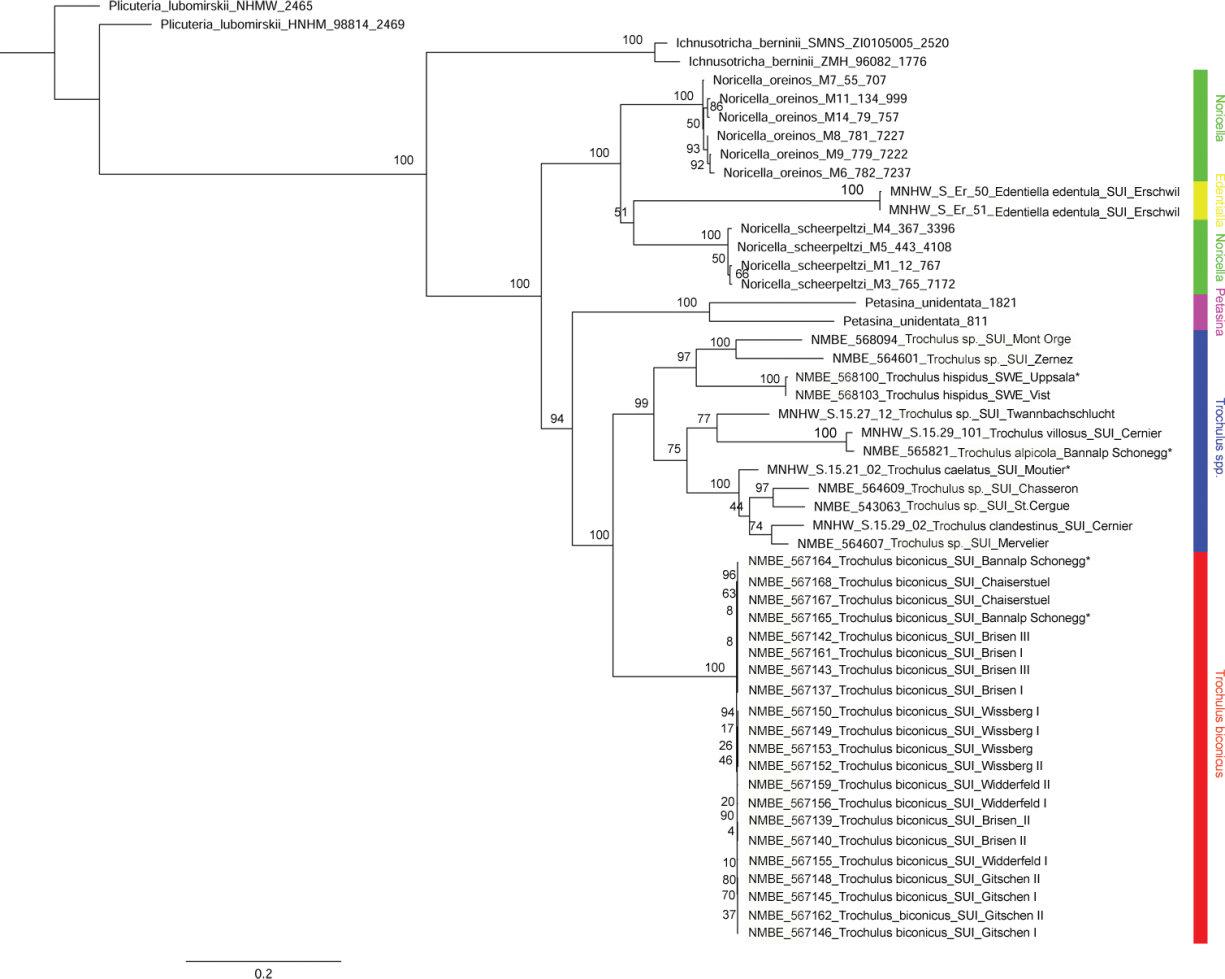
Maximum Likelihood (RAxML) tree based on the concatenated data set of COI, 16S, and 5.8S rRNA+ITS2. Numbers represent bootstrap support values from the ML analysis.

The ML analysis of the concatenated data set (Fig. 3) shows a similar topology as that of the BI analysis. The difference in the ML and the BI tree is the relationship of *Edentiella* and *Noricella*. In the ML tree, *E.edentula* clusters together with *N.scheerpeltzi*. This node has low support value (bootstrap support of 51 in Fig. 3), whereas in the BI analysis (Fig. 2), *Edentiella* and *Noricella* show a polytomy. In both analyses, *T.biconicus* forms the sister lineage to the selected *Trochulus* species. This node has full ML support. The support values within the *Trochulus* clade are moderate to high.

The *p*-distance, which shows the number of base differences per site from between sequences (Kumar et al. 2018) for the COI was calculated using MEGA v. 10.1.8 (https://www.megasoftware.net/). The *p*-distance for *T.biconicus* and the remaining investigated *Trochulus* species ranges from 0.153–0.189, for *T.biconicus* and *E.edentula* from 0.183–0.189, for *T.biconicus* and *Noricella* species from 0.128–0.166, for *T.biconicus* and *P.unidentata* from 0.171–0.176, for *T.biconicus* and *I.berninii* from 0.142–0.147 and for *T.biconicus* and *P.lubomirksi* from 0.177–0.188 (see Suppl. material 1). The genetic investigations in this study clearly show that *T.biconicus* is neither a member of the *Trochulus* clade nor does it belong to another known genus in the Trochulini. It thus, warrants designation in a separate new genus.

**Figure 4.**
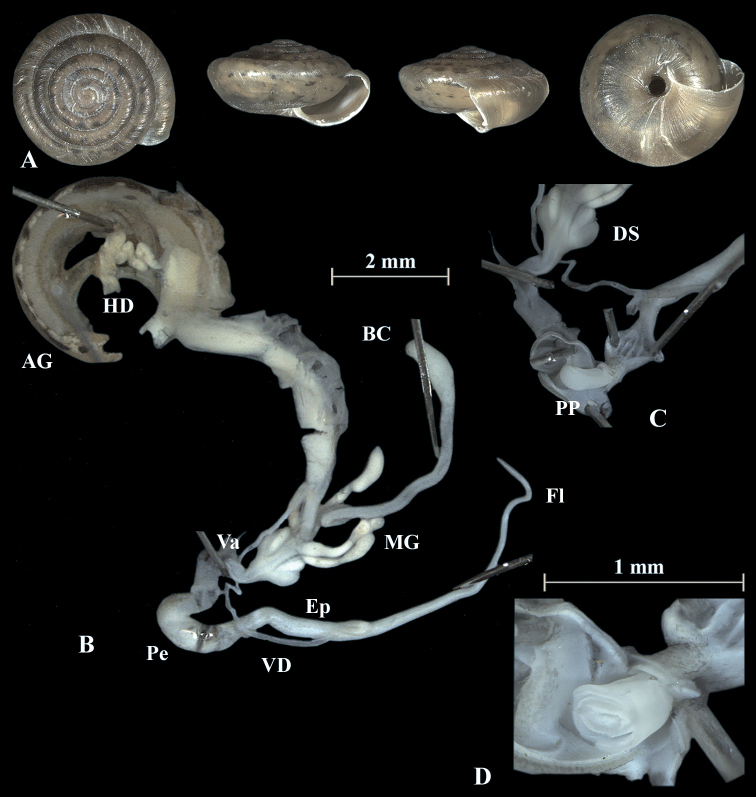
*Trochulusbiconicus* (NMBE 567151) collected from Wissberg I **A** shell, sw = 5.56 mm, sh = 2.55 mm **B** situs **C** penis (Pe) with penial papilla (PP) **D** cross section of the penial papilla. Shell × 5.

### ﻿Shell morphology

**Figure 5.**
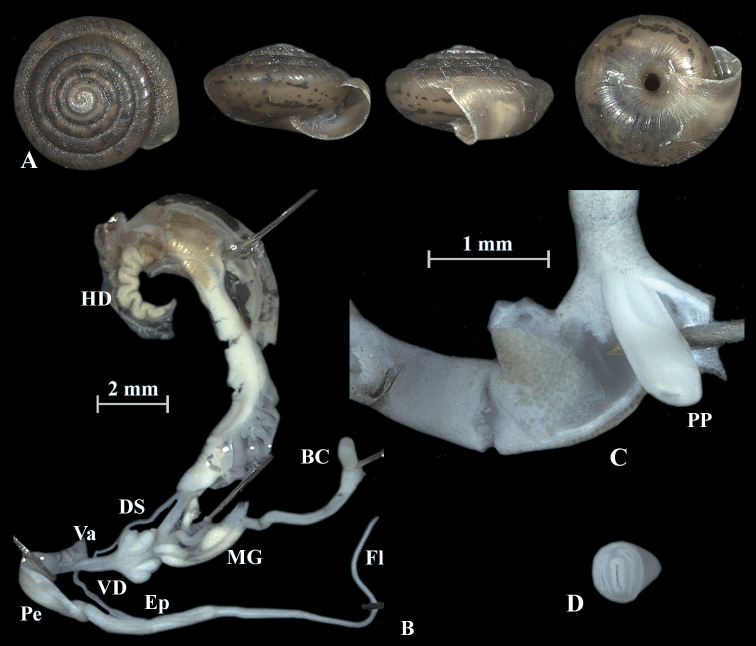
*Trochulusbiconicus* (NMBE 567160) collected from Widderfeld II **A** shell, sw = 5.73 mm, sh = 2.59 mm **B** situs **C** penis (Pe) with penial papilla (PP) **D** cross section of the penial papilla. Shell × 5.

**Figure 6.**
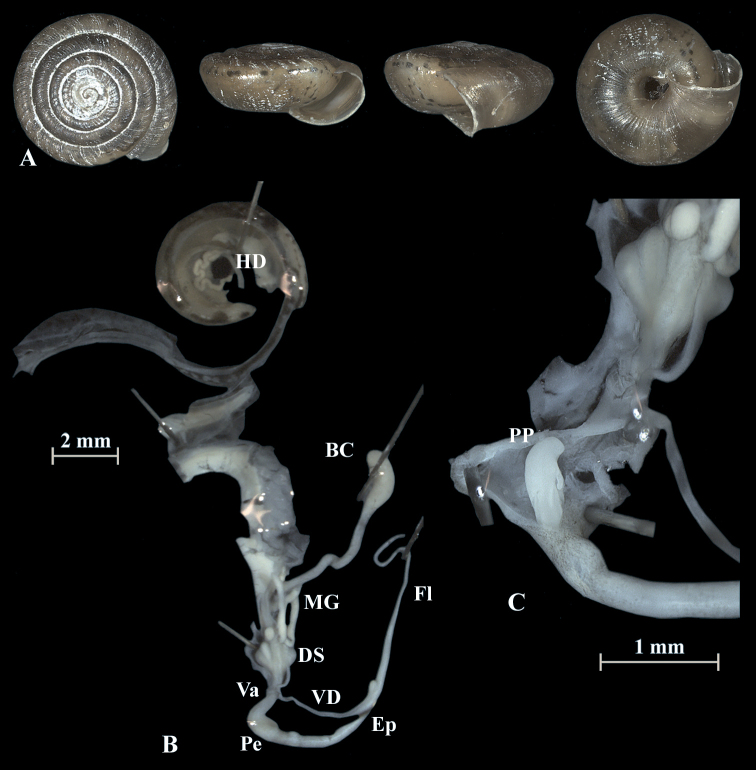
*Trochulusbiconicus* (NMBE 567138) collected from Brisen I **A** shell, sw = 5.61 mm, sh = 2.34 mm **B** situs **C** penis (Pe) with penial papilla (PP). Shell × 5.

**Figure 7.**
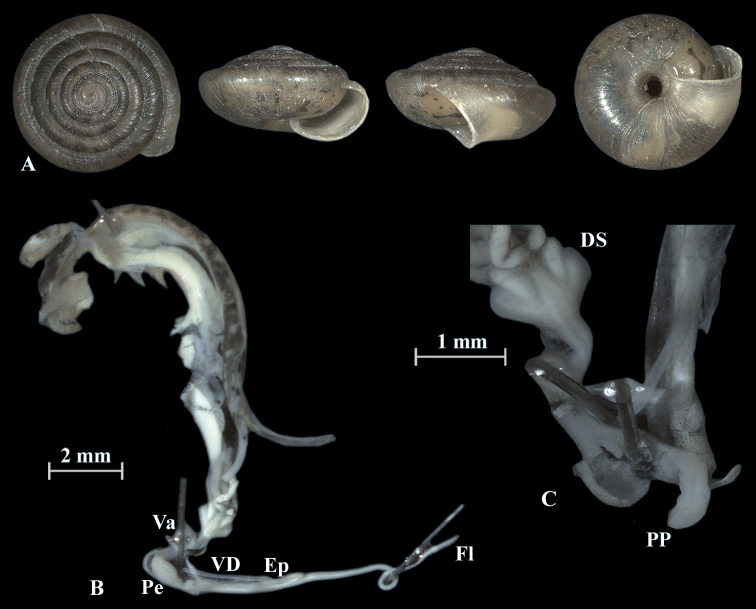
*Trochulusbiconicus* (NMBE 567163) collected from Gitschen II **A** shell, sw = 5.67 mm, sh = 2.87 mm **B** situs **C** penis (Pe) with penial papilla (PP). Shell × 5.

**Figure 8.**
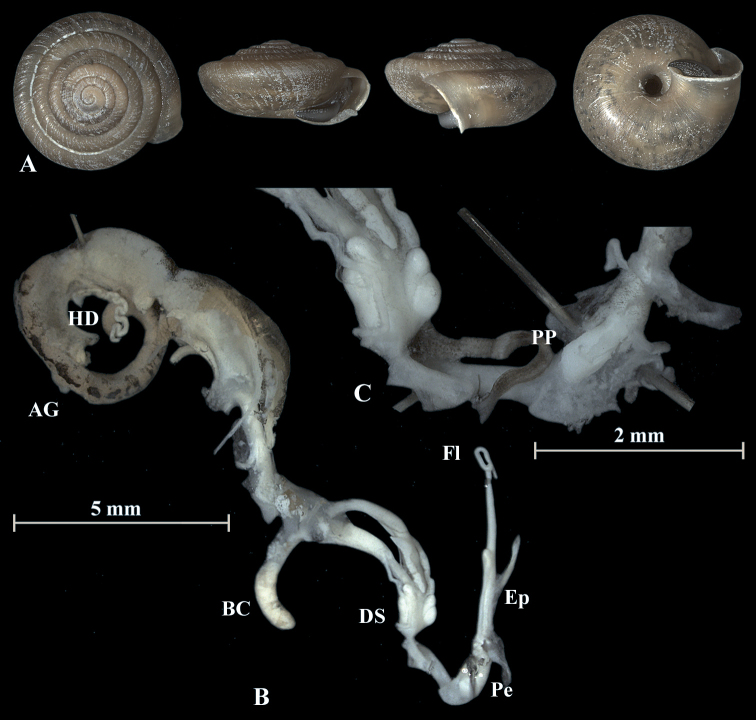
*Trochulusbiconicus* (NMBE 567166) collected from Bannalp Schonegg **A** shell, sw = 5.75 mm, sh = 2.76 mm **B** situs **C** penis (Pe) with penial papilla (PP). Shell × 5.

**Figure 9.**
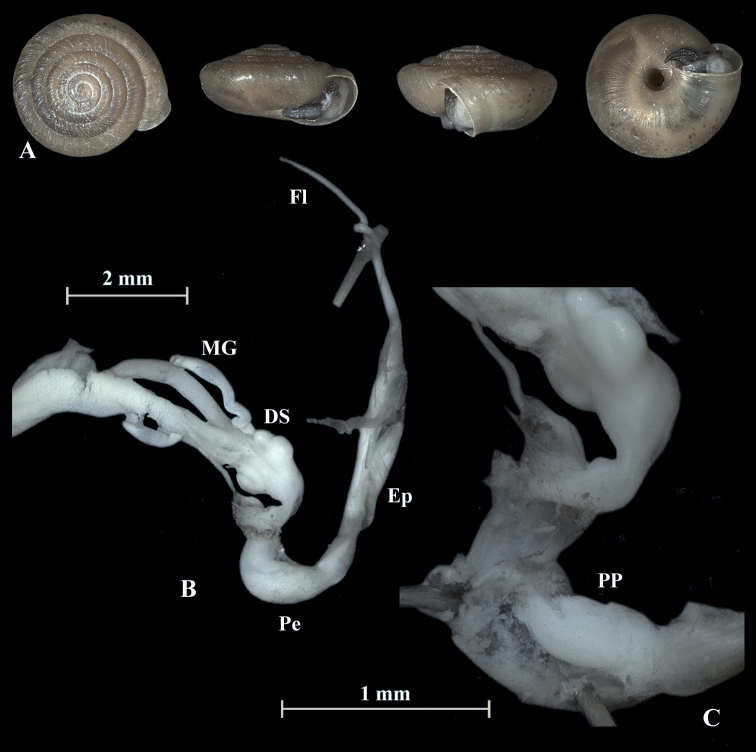
*Trochulusbiconicus* (NMBE 567169) collected from Chaiserstuel **A** shell, sw = 5.30 mm, sh = 2.46 mm **B** situs **C** penis (Pe) with penial papilla (PP). Shell × 5.

**Figure 10.**
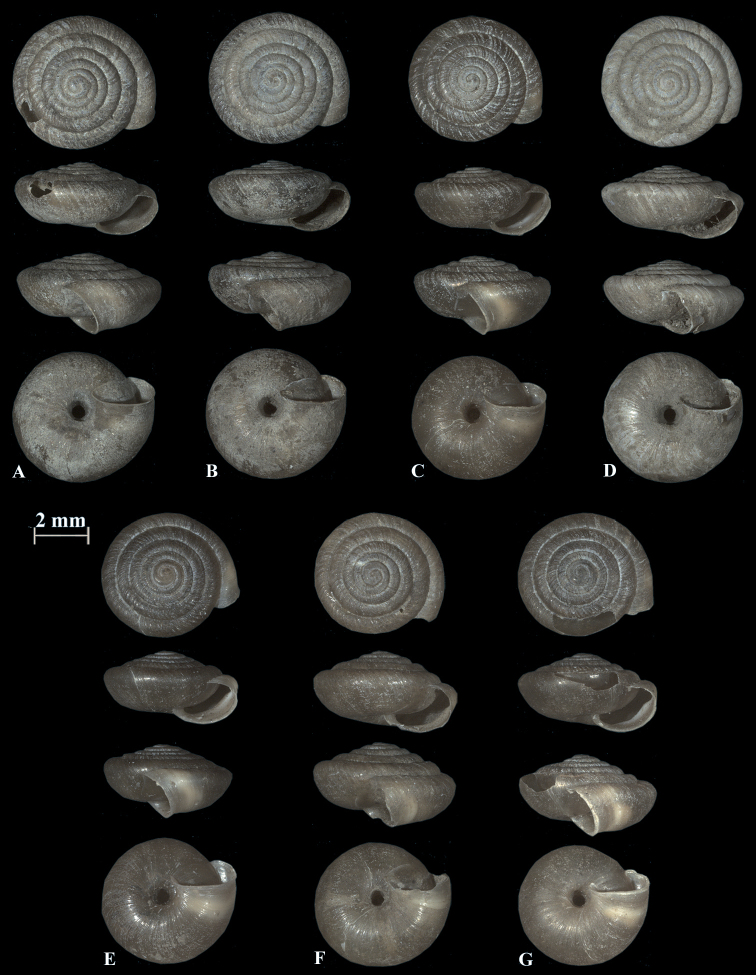
Shells of *Trochulusbiconicus* from Bannalp Schonegg (**A–D**) and from Chaiserstuel (**E–G**).

The shell of *T.biconicus* is flattened, tightly coiled, and beige to brownish. The mean shell width of the investigated individuals (*N* = 13) is 5.63 mm (range: 5.3–6.1 mm; SD = 0.23 mm) with mean shell height reaching 2.67 mm (range: 2.34–2.9 mm; SD = 0.17 mm) (Table 3). The shell bears 5.5–6 whorls which increase only slightly in width towards the perimeter. The umbilicus is entirely open and wide. The crescent-shaped aperture contains a white, poorly developed lip. Neither juveniles nor adults show hairs on the shell (Figs 4–10).

**Table 3.** Morphological analysis: measurements of the shell and genital organs of *T.biconicus* and *T.clandestinus*. Additionally, some collected dry shells from Bannalp Schonegg (NMBE 567170) and Chaiserstuel (NMBE 567171) were included in the analysis. Asterisk (*) marks the type locality of *T.biconicus*. Umbilicus minor diameter is measured according to Proćków (2009). All measurements are in mm.

Voucher No.	Species	Locality	Coordinates	Altitude [m]	shell width	shell height	umbilicus minor diameter	penis length	epiphallus length	flagellum length	Figure number
NMBE 567151	*T.biconicus*	Wissberg I	46.81°N, 8.47°E	2335	5.56	2.55	0.8	1.81	2.01	5.98	Fig. 4
NMBE 567160	*T.biconicus*	Widderfeld II	46.83°N, 8.33°E	2290	5.73	2.59	0.88	2.79	3.42	8.13	Fig. 5
NMBE 567138	*T.biconicus*	Brisen I	46.90°N, 8.45°E	2045	5.61	2.34	0.73	2.86	3.06	7.25	Fig. 6
NMBE 567163	*T.biconicus*	Gitschen II	46.88°N, 8.57°E	1970	5.67	2.87	0.84	2.23	3.67	6.38	Fig. 7
NMBE 567166	*T.biconicus*	Bannalp Schonegg*	46.87°N, 8.46°E	2232	5.75	2.76	0.96	1.84	1.98	4.26	Fig. 8
NMBE 567169	*T.biconicus*	Chaiserstuel	46.87°N, 8.46°E	2263	5.3	2.46	0.99	2.66	3.21	4.1	Fig. 9
NMBE 567170_1	*T.biconicus*	Bannalp Schonegg*	46.87°N, 8.46°E	2232	5.7	2.82	1.19	–	–	–	Fig. 10A
NMBE 567170_2	*T.biconicus*	Bannalp Schonegg*	46.87°N, 8.46°E	2232	6.03	2.73	1.08	–	–	–	Fig. 10B
NMBE 567170_3	*T.biconicus*	Bannalp Schonegg*	46.87°N, 8.46°E	2232	5.39	2.54	0.99	–	–	–	Fig. 10C
NMBE 567170_4	*T.biconicus*	Bannalp Schonegg*	46.87°N, 8.46°E	2232	6.1	2.9	1.07	–	–	–	Fig. 10D
NMBE 567171_1	*T.biconicus*	Chaiserstuel	46.87°N, 8.46°E	2263	5.46	2.76	1.08	–	–	–	Fig. 10E
NMBE 567171_2	*T.biconicus*	Chaiserstuel	46.87°N, 8.46°E	2263	5.41	2.61	0.79	–	–	–	Fig. 10F
NMBE 567171_3	*T.biconicus*	Chaiserstuel	46.87°N, 8.46°E	2263	5.46	2.83	0.89	–	–	–	Fig. 10G
NMBE 571318	*T.clandestinus*	Bern, Bümpliz	46.9435°N, 7.3922°E	540	9.64	5.57	1.29	4.24	5.79	4.69	Fig. 11

### ﻿Morphology of the genitalia

The genitalia are characterised by four stylophores, symmetrically placed in two pairs on both sides of the vagina (see fig. 11 in Proćków 2009). The inner dart sacs are somewhat longer and slenderer than the outer sacs. The outer stylophores contain the love darts (see also Proćków 2009). The mucous glands consist of four simple and thin tubes branching off the free oviduct directly above the dart sacs. The vagina is a rather long tube, which is almost smooth inside or shows some faint elongate tissue folds that connect to the atrium (not shown in the figures). The bursa copulatrix branches off from the free oviduct above the dart sacs and the mucous glands and is terminated by an elongated vesicle.

The penis is fusiform and contains a club-shaped penial papilla which points into the lumen of the penial chamber. The epiphallus is as long as the penis; the penis retractor muscle inserts at the transition zone between epiphallus and penis. The flagellum is about 1.5× the length of the penis and epiphallus each. The epiphallial lumen contains longitudinal tissue ridges (e.g., Fig. 4C). The penial chamber is characterised by smooth walls. The penial papilla contains a lateral subapical pore. The cross section of the penial papilla (Figs 4D, 5D) reveals a central duct surrounded by small folds.

**Figure 11.**
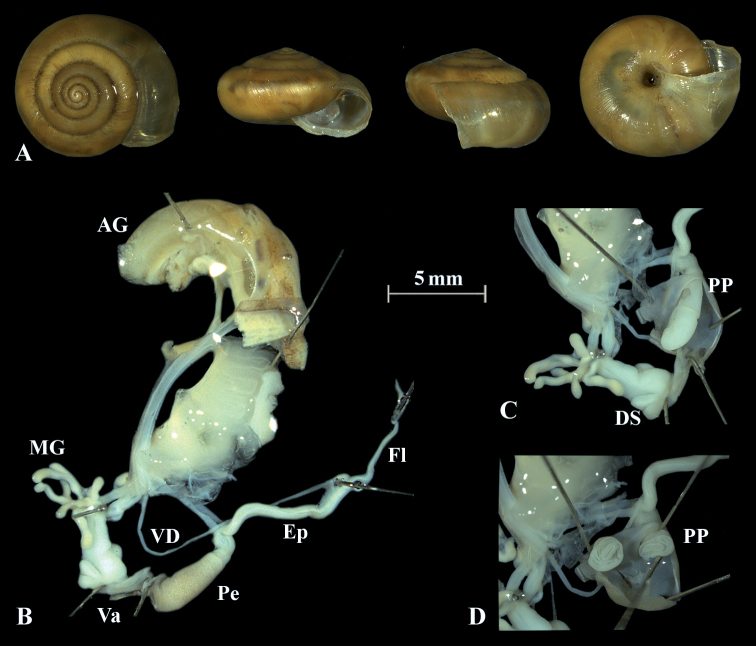
*Trochulusclandestinus* (NMBE 571318) collected from Bümpliz, Bern, Switzerland **A** shell, sw = 9.64 mm, sh = 5.57 mm **B** situs **C** penis (Pe) with penial papilla (PP) **D** cross section of the penial papilla. Shell × 3.

The anatomy of the genitalia of *T.clandestinus* differs from *T.biconicus* by having eight long, thin mucous glands (Fig. 11). The inner dart sacs of the investigated *T.clandestinus* are slightly longer in length than the outer dart sacs. The flagellum has about the same length as the bulbous penis, and the epiphallus is slightly longer than the penis. The cross section of the penial papilla differs in *T.clandestinus* by having several tissue layers around the main tube of the penial papilla (Fig. 11D).

### ﻿Taxonomic and systematic implications

The fully supported split between *T.biconicus* and currently known *Trochulus* species (Figs 2, 3) warrants description of a new genus, *Raeticella* gen. nov., based on *Fruticicolabiconica*.

#### ﻿Genus Raeticella gen. nov.

Taxon classificationAnimaliaStylommatophoraHygromiidae

A613490D-D161-5CB4-8C95-CCC0B5622FB7

http://zoobank.org/D7620E37-3AA3-45D2-BB3C-B55114AF36F2

##### Type species.

*Fruticicolabiconica* Eder, 1917.

#### ﻿Genus Trochulus

Taxon classificationAnimaliaStylommatophoraHygromiidae

Chemnitz, 1786

9E249B1E-F540-5203-B000-DD87278999DA

Trochulus biconicus (Eder, 1917)

##### Diagnosis.

Shell flattened and thin-walled, translucent, compressed in the direction of the axis; no trichome formation; whorls 5.5–6, gradually increasing so that the body whorl is only about twice as wide as the first whorl; the aperture is oblique, narrow, crescent-shaped; lip sharp, whitish and slightly reflexed; the four mucous glands are long, thick and pointed; penis and epiphallus are about the same length; the flagellum is barely separated from the epiphallus.

##### Differential diagnosis.

*Raeticella* gen. nov. differs from *Trochulus* by having a flat, biconical shell, devoid of any periostracal hairs, even in juveniles, and in having only four instead of occasionally six or eight (see Duda et al. 2014) mucous glands. It differs from *Noricella* by lacking a basal tooth, being devoid of any periostracal hairs, the absence of coarse ripples and the absence of an additional fold and bulge in the penial papilla, which occurs in *N.oreinos* (Duda et al. 2014).

##### Etymology.

The name is derived from the Roman province of Raetia, which comprised within its larger expansion, the area of what is now known as eastern and central Switzerland. It also refers to the generic name, *Noricella*, which is another recently detected spin-off from *Trochulus* and whose name derives in part from the eastern border province of Raetia (Noricum – now Austria and Slovenia).

## ﻿Discussion

Neiber et al. (2017) clarified the phylogenetic positions of some species within the Trochulini by establishing the new genus *Noricella* Neiber, Razkin & Hausdorf, 2017. In their study it was proven that *N.oreinos* and *N.scheerpeltzi* differed from the closest related genus *Edentiella* Poliński, 1929 in some apomorphic nucleotide substitutions and by morphological characters. *Edentiella* contains at least one longitudinal septum separating an additional lacuna in the penial papilla which is lacking in *N.oreinos*, in most *Trochulus* species, and in *Petasina* (Neiber et al. 2017). These authors also included some representatives of *Trochulus* but did not have specimens of *R.biconica* available. Turner et al. (1998) had already considered *R.biconica* to be only distantly related to *Trochulus* s. str. because of 1) the lack of periostracal hair even in juveniles, 2) a very long flagellum, and 3) only four instead of six or eight mucous glands. Hence, Turner (1991) suggested to move *R.biconica* into a subgenus of *Trochulus*. The questionable position of *biconicus* in *Trochulus* was recently re-addressed by Proćków et al. (2021). In our analysis, the calculated *p*-distance of *R.biconica* and the investigated *Trochulus* specimens comprises the highest values. The *p*-distance of *R.biconica* and *Noricella* species is lower than for *Trochulus*, which means that *Raeticella* is genetically closer, based on COI, to *Noricella* than to *Trochulus*. Even *Ichnusotricha*, which belongs to the tribe of Ganulini is genetically more similar to *Raeticella* than *Trochulus* is to *Raeticella*.

The shell morphology of *R.biconica* differs from all known *Trochulus* species by having a flat shell with a low spire. The last whorl is bluntly keeled. Adults are always hairless (Proćków 2009). In this regard, it is most like the shells of the two *Noricella* species (Duda et al. 2011, 2014), but the anatomy of the genital organs of these species is different. Both *Noricella* species have four pairs of mucous glands, compared to two pairs in *R.biconica*. *Noricellaoreinos* possesses an additional fold and bulge in the penial papilla, which seems to be unique to this species (Duda et al. 2014). The section of the penial papilla in *R.biconica* shows similar internal features as in *T.caelatus* (Proćków 2009), *T.striolatus* (Proćków 2009; Duda et al. 2014; Proćków et al. 2021), and *T.suberectus* (Proćków 2009). *Raeticellabiconica* does not possess periostracal hairs, neither as a juvenile nor as an adult. This, however, is considered a typical feature for *Trochulus* species (Proćków 2009).

Hewitt (2004) observed that many taxa in temperate refugial regions in Europe and North America show relatively deep DNA divergence, indicating their presence over several ice ages and suggesting a mode of speciation by repeated allopatry. On the one hand, this possibly explains the deep split between *Raeticella* and *Trochulus* and shifts the splitting event of these groups to the Pliocene. On the other hand, we observed a low genetic diversity within our analysed populations. So, this species probably underwent a bottleneck event during the Pleistocene and the Last Glacial Maximum (LGM). Some isolated populations obviously survived this icy period. The LGM lasted about 30–19 ka in the Alps. During that period, this area was covered by massive ice sheets, and the glaciers reached out to the forelands of both, the northern and southern side of the main alpine chains. However, mountain tops above more than 2000 m were not covered by ice during the LGM. The recession of the glaciers from their maximum extent started around 24 ka (see Ivy-Ochs 2015). We hypothesize that the original distribution area of *R.biconica* was much larger, but only a few individuals survived on neighbouring nunataks (glacial islands) during the LGM. A similar scenario is assumed for the evolution of the two *Noricella* species (Duda et al. 2011, 2014; Kruckenhauser et al. 2014). Gittenberger et al. (2004) also hypothesized the survival of *Ariantaarbustorumalpicola* (A. Férussac, 1821) on nunataks. A similarly fragmented distribution pattern can be observed in the eastern alpine mollusc species *Cylindrusobtusus* (Draparnaud, 1805) (Schileyko 2012: 95, fig. 2). Schileyko argued that the missing fossil record for this species proves that it was formed at the end of the Würm glaciation approximately 10–12 ka ago. As a species adapted to cold environmental conditions, this species was then assumed to be forced to follow the retreating snow and ice fields, which subsequently lead to habitat fragmentation. This assumption requires an ancestor from interglacials (which is also not found in the fossil record), and has to explain the rapid transformation of an Ariantine species from a globular or even depressed shell to a turriform shell. This is most unlikely. Based on COI sequences, Cadahía et al. (2014) estimated 1.5–12 mya for the split between *Arianta* and *Cylindrus*. So, we assume that *Raeticella* gen. nov., like the monotypic genus *Cylindrus*, evolved much earlier and survived the Pleistocene by chance on nunatak mountain tops.

The current distribution pattern does not necessarily and strictly reflect the “survivor” populations. ARNAL (2018) found a limited gene flow between the “isolated” populations of *R.biconica*. This shows that dispersal is not completely impossible, but, due to the high-altitude adaptation of the species, it is rather limited to other, hitherto unpopulated high alpine areas. Possible vectors may be large pasturing animals like sheep and goats, but also ibex, chamois, or birds.

In alpine environments, microendemic species with a relict distribution pattern may occur, which were much more widespread in earlier times. They are now restricted to a very small area due to changes in environmental condition (Turner 1991; Cook 2008; Veron et al. 2019). The distribution area of *R.biconica* is currently known to encompass 150 isolated sites on both sides of the Engelberger valley, all situated between 1890 and 2575 m of altitude (Baggenstos 2010).

**Figure 12.**
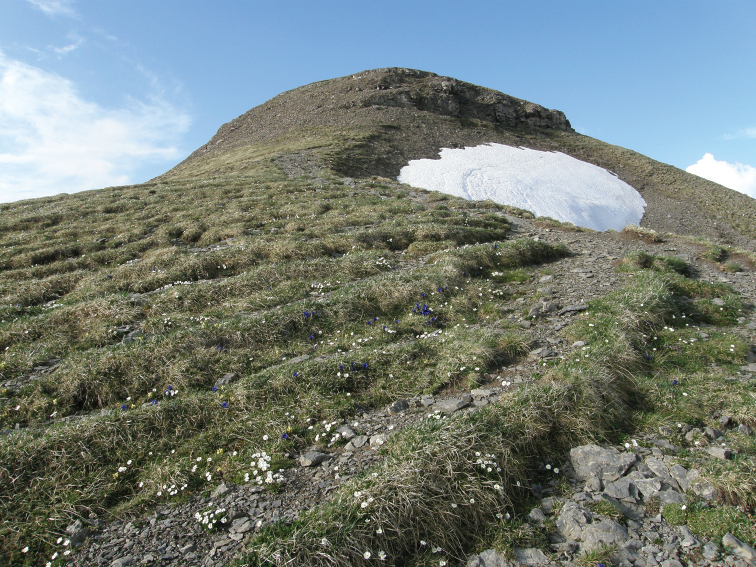
Typical habitat of *Raeticellabiconica*. This photograph was taken on 25.05.2009 on Chaiserstuel (46.8762°N, 8.4671°E, 2263 m) by Markus Baggenstos.

**Figure 13.**
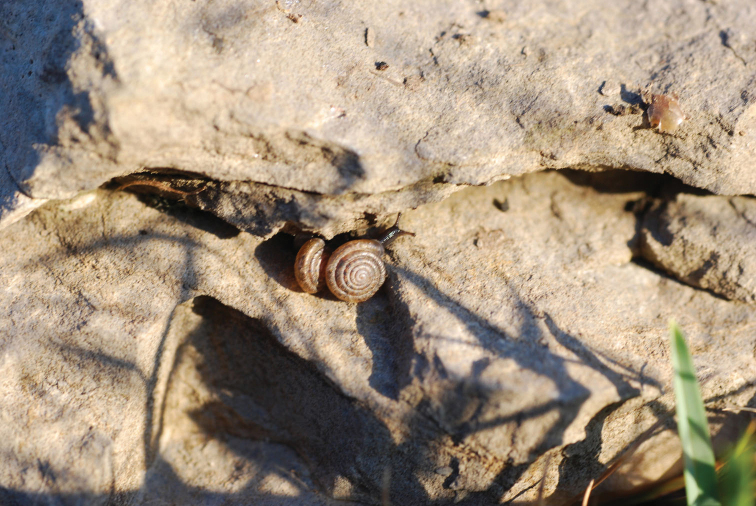
Close-up of *Raeticellabiconica* crawling on the underside of a stone. This photograph was taken on 09.09.2009 on Chaiserstuel (46.8762°N, 8.4671°E, 2263 m) by Markus Baggenstos.

The habitat of *R.biconica* is very special, and only few other snail species are known to survive in this harsh environment (Eder 1917; Baggenstos 2010). Apart from the occurrence of limestone scree, the snails very much depend on small-scale relief. Slope edges or hilltops, ridges and summits as well as rocky heads and rocky steps are more likely to be colonised by the snail than slope hollows and slope foothills. The highest density of *R.biconica* is reached in areas with more than 50% of rocky scree (Baggenstos 2010). All these sites are covered with snow for a relatively short time in winter. With its flat shell, *R.biconica* is perfectly adapted to live under or between stones (Figs 12, 13). Flatness was interpreted as an adaptation to the cold climate at the top of the mountains and may protect the animals from predators (Baur 1987). When it gets too hot, the snails retreat into the ground. The individuals are mainly active during night (Baggenstos 2010). Almost all known *R.biconica* habitats are blue grass meadows. These are alpine grasslands rich in flowers with a great diversity and a remarkably high proportion of Leguminosae. The prominent structural elements are *Sesleriacaerulea* and *Carexsempervirens*. The soil cover is relatively thin, interspersed with gravel and stones and dries out quickly (Delarze et al. 2008). Wigger (2007) observed that *R.biconica* mainly feeds on decaying leaves of blue grass (*Sesleriacaerulea*). The landscape of these meadows is strongly influenced by extensive pasturing and hiking tourism. Pasture animals like sheep, goats, and cows can modify the position of large stones and thus create new micro habitats for the snails. However, stronger interventions, such as the removal of stones or a climate-related transfer of the rubble-rich sites into closed meadows or woodland formations would cause the snail to disappear (Turner 1991).

This stenoecious species is prone to extinction because of climate change. Over the last 100 years temperatures have increased by about 0.12–0.20 °C per decade in the Swiss Alps and the snow seasons have shortened (Kohler et al. 2014). *Raeticellabiconica* already reached the summits of the mountains in their vicinity, and there is no more alternative for avoiding unsuitable climate conditions. Considering that global warming is ongoing, *R.biconica* may well become extinct in just a few years.

## ﻿Conclusion

Long known morphological characteristics in conjunction with our genetic analyses show that *R.biconica* should be assigned to a new genus. Morphologically, the investigated individuals of *R.biconica* strongly resemble *N.oreinos* (Duda et al. 2011). But the genetic analyses of several different species from all genera within Trochulini reveal that *R.biconica* does not belong to any currently known genus. Therefore, a new monotypic genus within Trochulini is introduced.

## Supplementary Material

XML Treatment for Raeticella

XML Treatment for Trochulus

## Supplementary materials

Supplementary material 1 Calculated p-distances of the COI of the investigated specimens. Data type excel file Explanation note Calculated p-distances of the COI of the investigated specimens. https://binary.pensoft.net/file/693633This dataset is made available under the Open Database License (http://opendatacommons.org/licenses/odbl/1.0/). The Open Database License (ODbL) is a license agreement intended to allow users to freely share, modify, and use this Dataset while maintaining this same freedom for others, provided that the original source and author(s) are credited.Jeannette Kneubühler, Markus Baggenstos, Eike Neubert
